# Multifocal Lupus Vulgaris: A Rare Presentation

**DOI:** 10.7759/cureus.40495

**Published:** 2023-06-16

**Authors:** Nidhi Pugalia, Bhushan Madke, Sugat Jawade

**Affiliations:** 1 Dermatology, Venereology and Leprosy, Jawaharlal Nehru Medical College, Datta Meghe Institute of Higher Education and Research, Wardha, IND

**Keywords:** mycobaterium, disseminated tuberculosis, cutaneous tb, multifocal, lupus vulgaris

## Abstract

Lupus vulgaris (LV) is a common type of cutaneous tuberculosis and commonly presents as a single erythematous plaque either on the face or buttocks with scarring and an active spreading edge. Multiple lesions of LV are sparingly reported in the literature. We hereby report a case of LV in a male presenting with multiple lesions over the buttock, thigh, and trunk. The diagnosis was done on the basis of clinical findings, histopathology, positive tuberculin test, and response to a standard anti-tubercular regimen.

## Introduction

Cutaneous tuberculosis (TB) accounts for 2% of TB cases [1]. Scrofuloderma (50%), lupus vulgaris (LV) (42.86%), TB verrucosa cutis (4.76%), and lichen scrofulosorum (2.38%) are the most prevalent types of cutaneous TB in India [1]. Females are at greater risk of getting LV and scrofuloderma as compared to males, who are more likely to acquire TB verrucosa cutis and ulcerative TB [2]. In India, scrofuloderma is the most common type of cutaneous TB in children, whereas the most prevalent form in adults is LV [3]. LV is a chronic, progressive, paucibacillary form of secondary cutaneous TB, with a female preponderance in a previously sensitized host having moderate to high immunity and a high degree of delayed hypersensitivity to tuberculin [1].

## Case presentation

**Figure 1:**
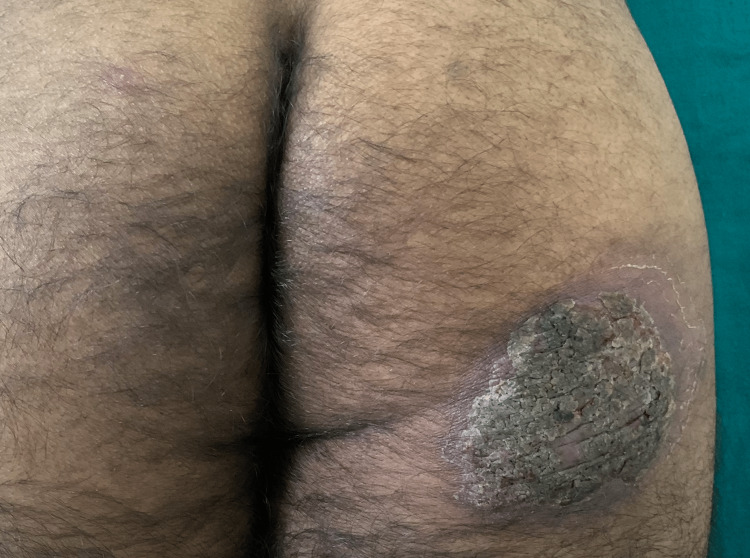
Well-defined, hyperkeratotic plaque of lupus vulgaris with irregular margins over the right buttock.

**Figure 2:**
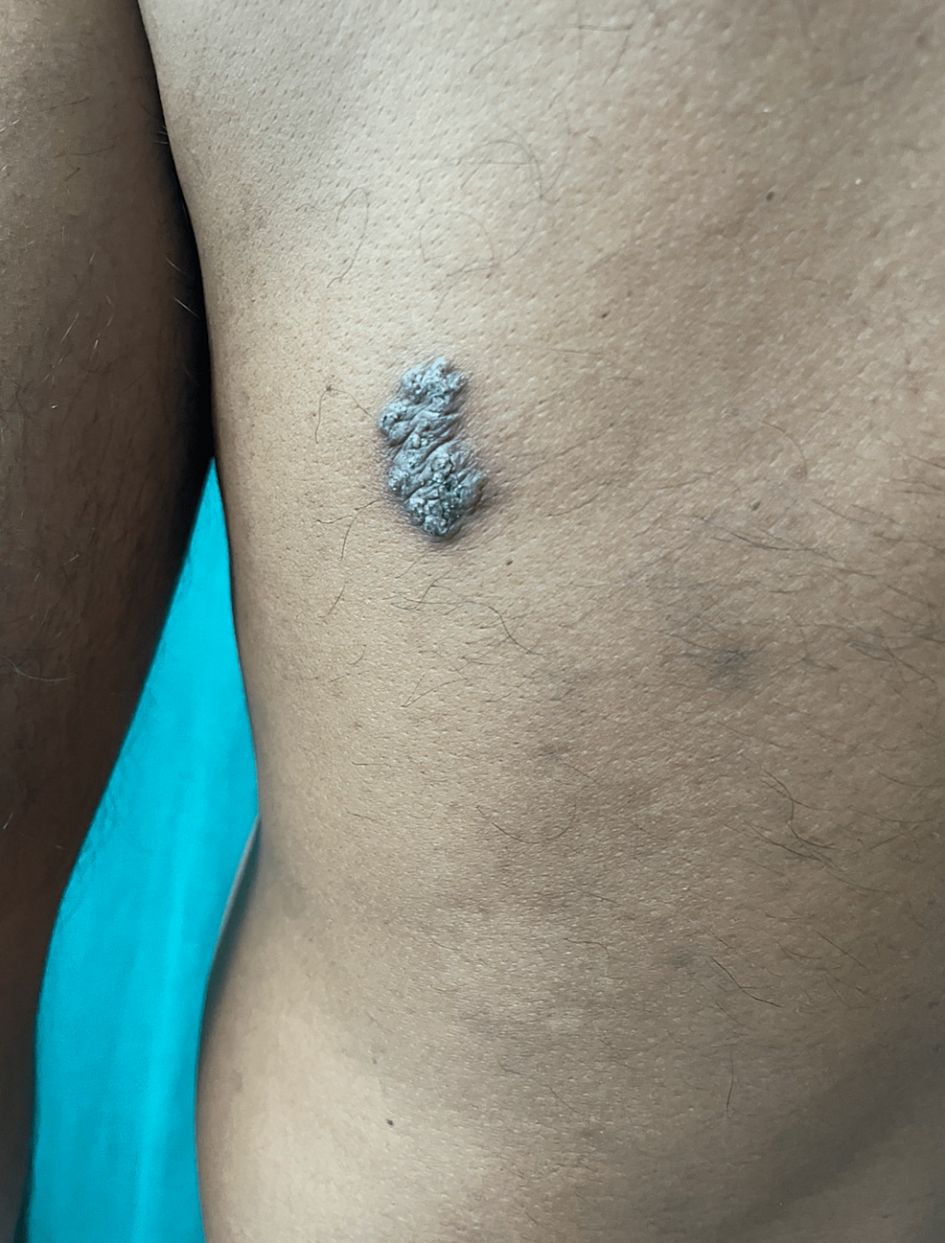
Well-defined, hyperkeratotic, verrucous, plaque of lupus vulgaris with irregular margins over the right side of the trunk.

**Figure 3:**
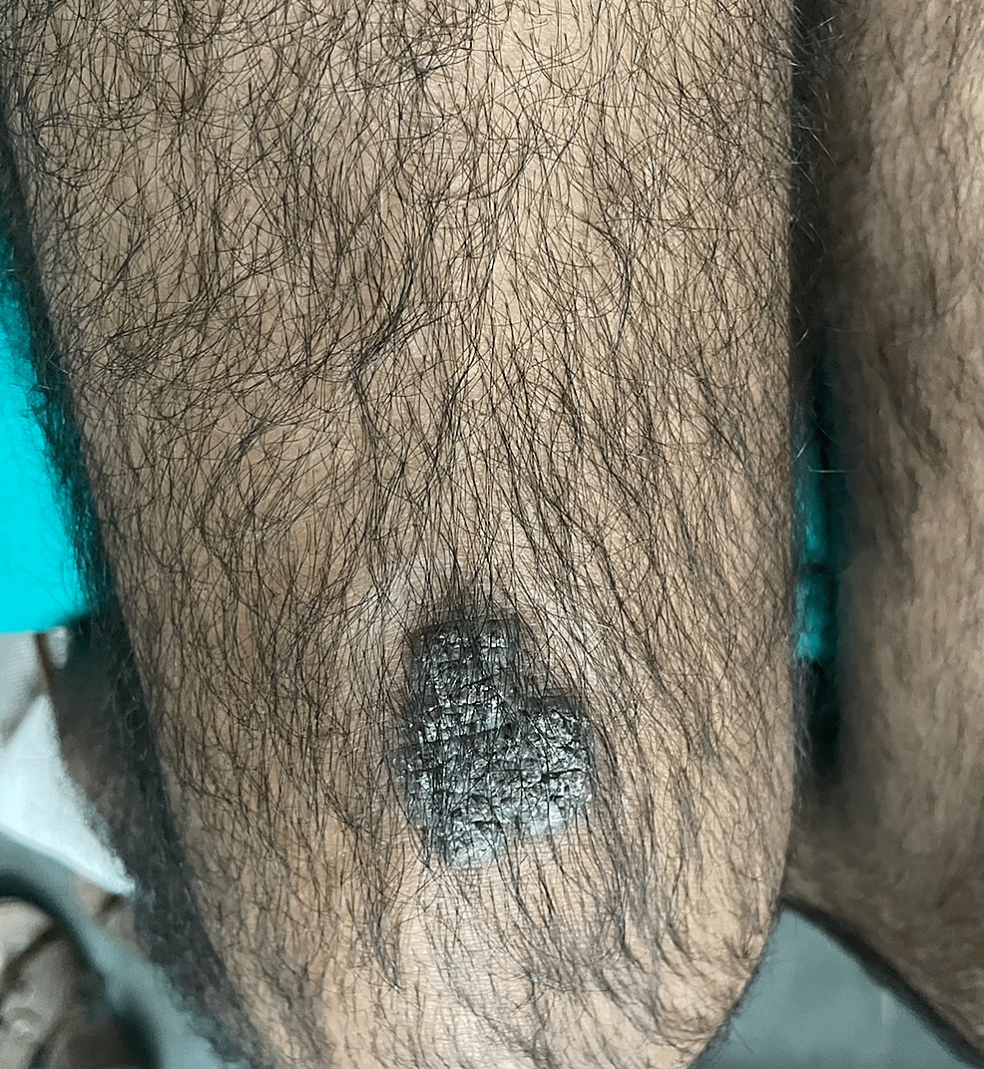
Well-defined, hyperkeratotic, dusky, plaque of lupus vulgaris with irregular margins over the anterior aspect of the left thigh.

**Figure 4:**
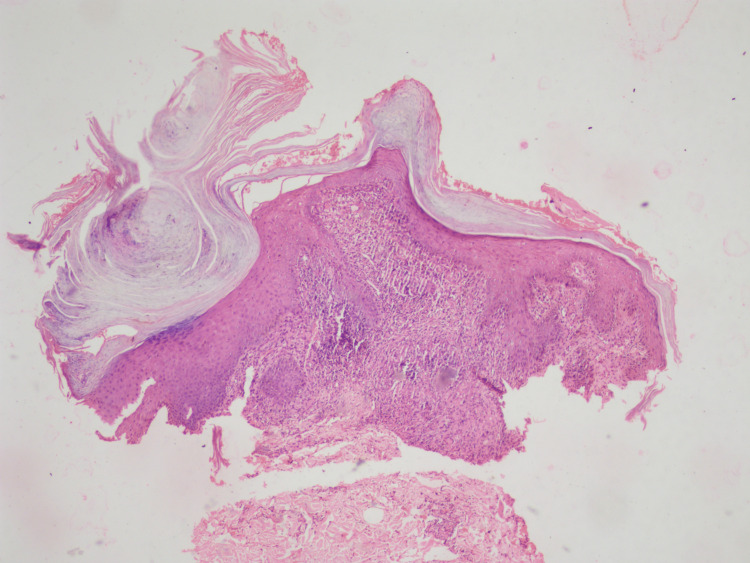
Histopathological section with h and e stain (4x scanner view) showing compact orthokeratosis, parakeratosis, moderate acanthosis profuse lichenoid granulomatous infiltration in the dermis with few foreign body giant cells in the upper reticular dermis.

**Figure 5:**
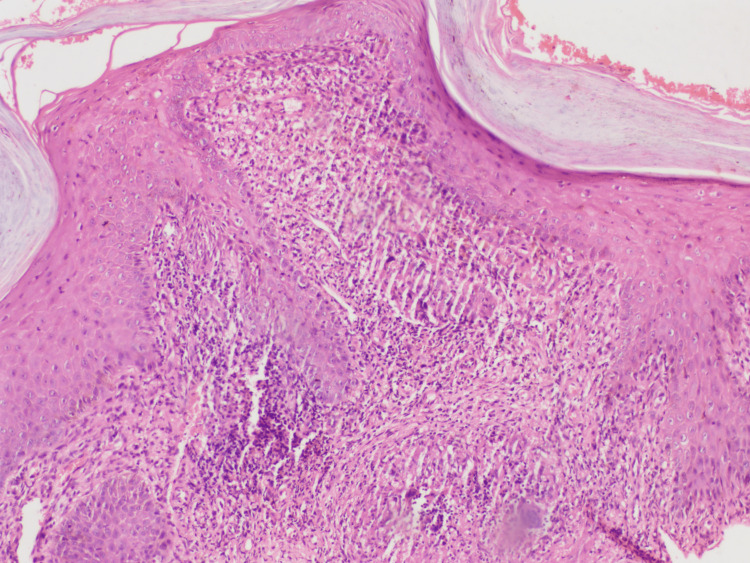
Histopathological section with H and E stain (10x view) showing compact orthokeratosis, parakeratosis, moderate acanthosis profuse lichenoid granulomatous infiltration in the dermis with few foreign body giant cells in the upper reticular dermis.

**Figure 6:**
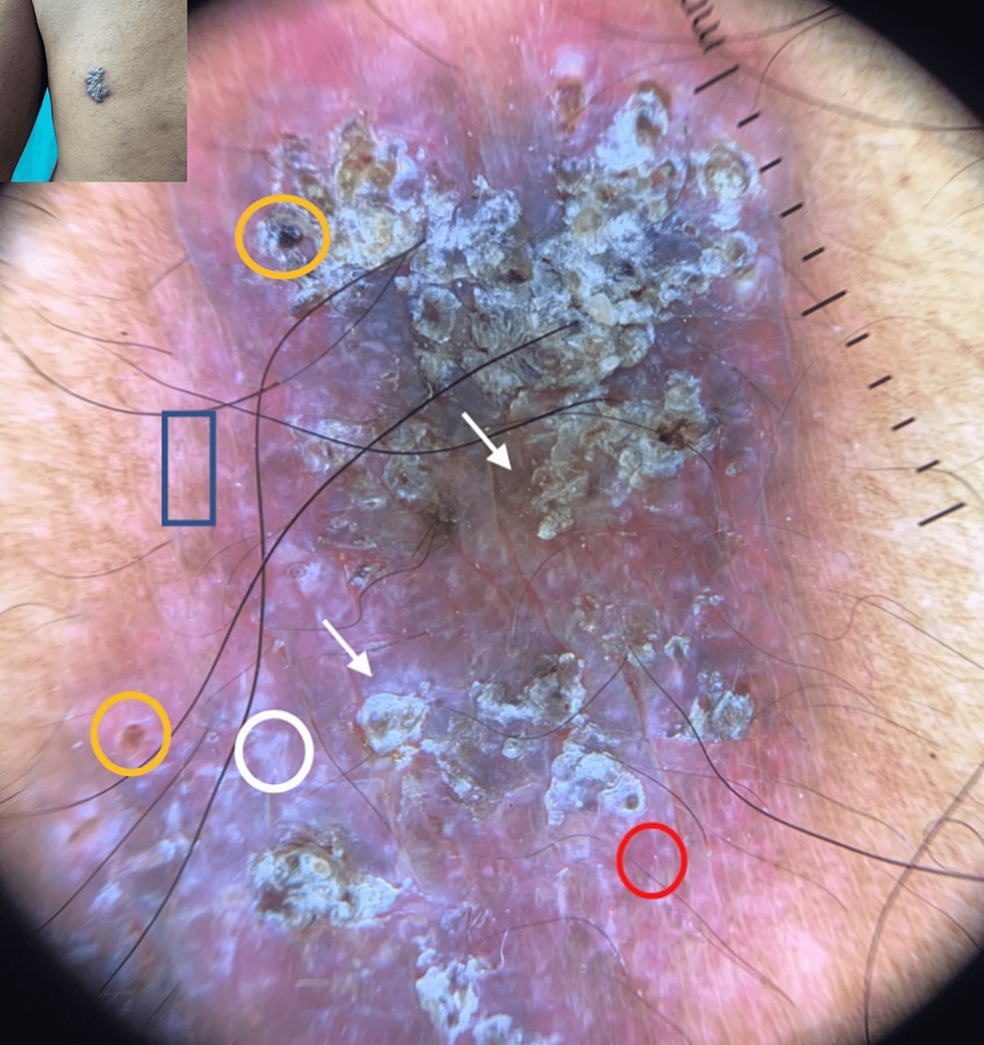
Dermoscopy (Dermlite 4) revealing white structureless areas of keratin and collagen (white circle), follicular plugging (yellow circle), peri-lesional halo (blue rectangle), red globules of elongated papillary loops over pinkish background (red circle), white-yellow scaling (white arrowhead) and bluish hue of orthokeratosis.

A 38-year-old immunocompetent male presented to an out-patient section of a dermatology clinic with asymptomatic, non-healing plaque over the right side of the trunk, right buttock and left thigh for the past two years. All three lesions started as a small papule and slowly increased in size over a period of two years. There was a history of occasional pruritus over the lesions. Cutaneous examination showed three verrucous hyperkeratotic, dusky red plaques with irregular regions of atrophy, and adherent yellow-brown crusts over multiple sites. There were three plaques with well-defined borders and irregular margins. Lesion on the right buttock, measured about 15 cm x 10 cm (Figure 1), one on the right side of the trunk was about 3cm × 5 cm (Figure 2), while the lesion on the anterior aspect of the left thigh measured about 7cm x 4 cm (Figure 3). There was no past history suggesting pulmonary or visceral tuberculosis in the patient or in close contact. There was no significant enlargement of draining lymph nodes. Complete general and systemic examinations were normal. Complete hemogram, liver and renal function tests were within normal range. The Tuberculin skin test was positive (12mm). Cartridge-based Nucleic Acid Amplification Test (CBNAAT) was negative and chest radiography did not show any lung or bony pathology suggestive of tuberculosis. A punch biopsy from one of the lesions showed compact orthokeratosis and parakeratosis with moderate acanthosis. The dermis showed lichenoid granulomatous infiltration comprising lymphocytes, histiocytes, and plenty of plasma cells occupying the papillary and mid-reticular dermis. Foreign body giant cells were seen at a few places in the upper reticular dermis (Figures 4, 5). Standard anti-tubercular treatment was commenced. A dermoscopy of the lesion done with a polarized dermoscope (Dermlite DL4) performed showed white structureless areas corresponding to keratin and collagen, follicular plugging, peri-lesional halo, red globules on pinkish background implying elongation of papillary loops and vasodilation, hyperkeratosis, profuse scaling, and bluish hue indicating orthokeratotic areas (Figure 6).

 

## Discussion

Cutaneous TB can be classified as multibacillary (tuberculous chancre, acute military TB, scrofuloderma) and paucibacillary (tuberculids, LV and TBVC). Clinical presentation depends on the portal of entry of bacilli, host immunity status, and antimicrobial resistance [1,2]. LV is usually thought to arise from an underlying focus of either lymph node, bone or joint TB (usually or lymph node, joint, bone) or due to reactivation of latent cutaneous focus secondary to previous silent bacteremia or from direct inoculation (especially at the site of BCG vaccination) or lympho-hematogenous extension, that is both, exogenous and endogenous routes, respectively [3]. In the Western World, 80% of cases of LV occur on the head and neck, in particular, around the nose, followed by arms and legs, but trunk involvement is uncommon. While buttocks and extremities are commonly involved in the Indian subcontinent and can be explained by the risk of traumatic inoculation during outdoor work in the Indian subcontinent [3-5]. Atrophic scarring is a characteristic feature of LV, which can occur with or without ulceration. When the lesions are blanched by diascopy, they show a light brownish-yellow or “apple-jelly” hue that is distinctive to the disease. Fine-focused telangiectasias on a yellow to the golden background have been described as the typical dermoscopic findings of LV and are correlated with the apple-jelly sign [6]. “Lupoma” is a characteristic red-brown papule with a soft consistency, frequently localized on the head and neck region in LV [2]. Being a paucibacillary infection, tissue staining with acid-fast stain rarely shows the presence of bacilli [7]. Depending on the local tissue response to infection, clinical variants of LV are categorized into five different patterns: (a) Plaque form. (b) Ulcerative and mutilating form. (c) Vegetating form. (d) Tumor-like (Hypertrophic) form. (e) Papulo- nodular form. Basal cell carcinoma (BCC) and squamous cell carcinoma (SCC) are implicated in about 8% insidious cases which might be mistaken for reactivated lupus [3]. Dermoscopy of the LV can aid in the diagnosis. Ankad has proposed dermoscopic findings in LV [8]. Dermoscopic features in LV are summarized in Table 1.

**Table 1:** Dermoscopic findings and histopathological correlation of lupus vulgaris.

Dermoscopic pattern	Histopathological correlation
Yellowish-white globules	Dermal granulomas
Pinkish-red background	Widespread vasodilatation
Dotted vessels	Tips of normal vertical papillary loops
Red globules	Tips of ectatic/elongated papillary loops
Linear vessels	Ectatic/elongated subpapillary capillary plexus
Ulceration	Erosions in the epidermis and dermis
Whitish structureless areas	Acanthosis and dermal fibrosis White structureless area correlate with keratin (acanthosis) and collagen (fibrosis)
Superficial white or yellow scales	Hyperkeratosis and/or parakeratosis
White shiny streaks	Orientation of collagen bundles in different angles in the dermis
Follicular plugs	Keratotic material in follicular infundibulum
Patulous follicles	Dilatation of infundibulum
Bluish hue	Orthokeratosis

## Conclusions

To conclude, we report a case of multifocal LV in a male which in India, shows a slight female preponderance. we emphasize its presentation as multiple lesions including an uncommon site (trunk) while lupus usually presents as a singular lesion. We based our diagnosis on typical clinical appearance, histopathology, and positive tuberculin test.
